# abCRISPR: deep learning-based design of abasic gRNA sequences for specific CRISPR-Cas9 genome editing

**DOI:** 10.1093/bioinformatics/btag568

**Published:** 2026-07-29

**Authors:** Geun-Woo D Kim, Dowoon Gu, Mingyo Park, Sung Wook Chi

**Affiliations:** KU-KIST Graduate School of Converging Science and Technology, Korea University, Seoul 02841, Korea; Department of Life Sciences, Korea University, Seoul 02841, Korea; Department of Life Sciences, Korea University, Seoul 02841, Korea; KU-KIST Graduate School of Converging Science and Technology, Korea University, Seoul 02841, Korea; Department of Life Sciences, Korea University, Seoul 02841, Korea; National Research Laboratory for Convergence Degradation Biology, Korea University, Seoul 02841, Korea; Medicinal Materials Research Center, Biomedical Research Division, Korea Institute of Science and Technology (KIST), Seoul 02841, Korea

## Abstract

**Summary:**

CRISPR-Cas9 has become a widely used tool for genome editing. However, its off-target cleavage caused by partial sequence matches with guide RNAs (gRNAs) remains a critical limitation. Recently, abasic gRNAs (ØXØ) have been developed to enhance target specificity, but their effects vary depending on the positional sequence context. Here, we present abCRISPR, a deep neural network (DNN) framework for the rational design of ØXØ sequences with minimized off-target activity. abCRISPR leverages informative few-shot training with paired datasets of abasic and unmodified gRNAs, using high-quality random mismatch target libraries, exhaustively sequenced for mismatched off-target substrates (*n *= 97583) in *in vitro* CRISPR-Cas9 cleavage experiments. Predicted off-target activities for both abasic and unmodified gRNAs showed strong correlation with experimental data (*r *≥ 0.95, 10-fold cross-validation). Notably, these comprehensive training sets provide robust ground-truth negatives, enabling accurate and sensitive prediction of off-targets. For unmodified gRNAs, abCRISPR (AUC = 0.98) was validated to outperform existing deep learning-based methods (AUC = 0.45–0.68). When applied to the human genome, abCRISPR generated ØXØ sequences, covering 58 875 004 potent CRISPR-targetable sites with improved target specificity. Together, this work provides a comprehensive bioinformatics resource for safe and precise CRISPR-Cas9 genome editing.

**Availability and implementation:**

The source code for abCRISPR and training data are available at https://doi.org/10.5281/zenodo.20398246. abCRISPR results for the human genome are available at http://clip.korea.ac.kr/abCRISPR/

## 1 Introduction

**Figure 1:**
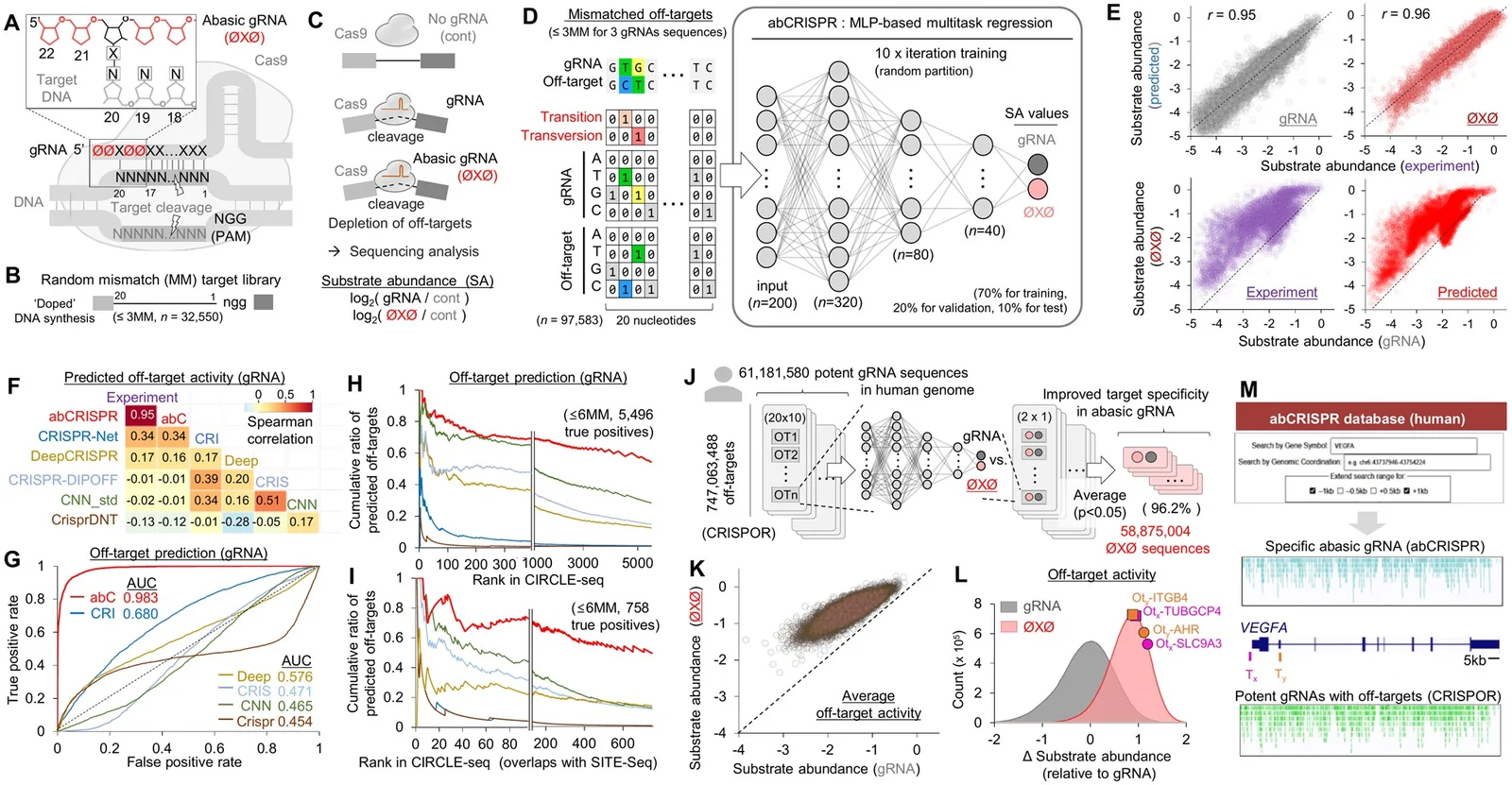
Overview and performance of abCRISPR to design abasic (ØXØ) gRNAs for specific CRISPR-Cas9 genome editing. (A) Schematic of ØXØ gRNA in CRISPR-Cas9-mediated cleavage for genome editing; Ø, abasic dSpacer. (B, C) Schematic of a random mismatch target library with up to 3 mismatches (≤3MM), generated by pooling 20 distinct “doped” synthetic DNAs (B; details in Fig. 1A, available as supplementary data at *Bioinformatics* online) and used to generate training sets derived from *in vitro* Cas9 cleavage experiments (C) that quantify depletion of off-target sequences with ØXØ gRNA versus unmodified gRNA (substrate abundance, SA; log_2_ ratio relative to no gRNA control). (D) Schematic of abCRISPR to predict SA values for ØXØ gRNA and unmodified gRNA, constructed using multilayer perceptron (MLP)-based multitask regression model with three hidden layers, considering a 20-nt-long spacer with dinucleotide context (2-nt; *n *= 4 × 4 × 20), four bases (4-base; *n *= 4 × 20), and match/mismatch Boolean (mismatch; *n *= 2 × 20). The model was trained with sequences (gRNA and off-targets), encoded considering transition/transversion mismatch within a 20-nt-long spacer, and SA values (70% for training, 20% for validation, and 10% for testing; across 10 iterations). (E) 10-fold cross-validation results from the abCRISPR model; scatter plots of SA values between prediction and experiment for the unmodified gRNA and ØXØ gRNA (upper panels); scatter plots of SA values between unmodified gRNA and ØXØ gRNA, compared between experiment and prediction (lower panels); Pearson correlation coefficient, *r*. (F, G) Pairwise correlation matrix (Spearman correlation coefficient; F) of predicted off-target activities and receiver operating characteristic (ROC; G) curves of off-target prediction between different deep-learning-based models including abCRISPR in 10-fold cross-validation; area under the curve (AUC) values. Off-targets were defined by an SA threshold of −2.17 (derived from CRISPR-Cas9 experiments; Fig. 1C and D, available as supplementary data at *Bioinformatics* online). (H) Cumulative fraction of predicted off-targets from different deep-learning-based models including abCRISPR in true positives identified by CIRCLE-seq (≤6MM) depending on read-count rank, indicating the outperformance of abCRISPR (SA < −2.16, determined as the highest F1 score; other models, the developer-used threshold of 0.5) with sustained superior positive predictive values. (I) Same analysis as in (H) except using a CIRCLE-seq dataset that overlaps with the SITE-Seq dataset. (J–M) Application of abCRISPR to the human genome (hg19) to design ØXØ gRNA with enhanced target specificity, predicting SA values from putative off-target sequences (*n *= 747 063 488; ≤3MM) of potent gRNAs (*n *= 61 181 580; efficiency > 55 and specificity > 50 in CRISPOR). 96.2% of ØXØ gRNAs resulted in significant increase in SA values for off-targets (*n *= 58 875 004; *P *< .05, relative to unmodified gRNA), indicating improved target specificity (J). A scatter plot of average SA values between ØXØ gRNA and unmodified gRNA (K). Distribution of SA value differences (L) between ØXØ gRNA and unmodified gRNA for the putative off-target sequences (Δsubstrate abundance, relative to the median of the unmodified gRNAs), indicating experimentally validated off-target sites (circle and rectangle), each labelled in the panel. The abCRISPR web-interface (e.g. VEGFA) supports searches by gene symbol or genomic coordinates (hg19) with user-selected flanking windows (±0.5 or ±1 kb; top panel) and returns a list of designed abasic gRNAs with links to the UCSC Genome Browser (M; https://genome.ucsc.edu/cite.html); abCRISPR track (upper) and CRISPOR track (lower); on-target sites of Tx and Ty are indicated.

The clustered regularly interspaced short palindromic repeats (CRISPR)-associated protein 9 (Cas9) system is a widely used genome editing tool (Doudna and Charpentier 2014, Hsu *et al.* 2014, Sander and Joung 2014). The CRISPR-Cas9 system can be easily programmed to cleave any DNA sequence by designing a spacer sequence in the guide RNA (gRNA) complementary to a 20-nucleotide (nt) target site (positions 1–20) adjacent to an NGG protospacer adjacent motif (PAM) (Cho *et al.* 2013, Cong *et al.* 2013, Mali *et al.* 2013). Nevertheless, serious concerns remain as it can also induce cleavage at numerous mismatched off-target sites (up to six mismatches; ≤6MM) through partial base pairing of the gRNA with genome sequences (Fu *et al.* 2013, Hsu *et al.* 2013, Lin *et al.* 2014, Zhang *et al.* 2015). Recently, based on the observation that abasic oxidation of CRISPR RNA naturally reduces off-target activity in *Streptococcus pyogenes* during bacteriophage infection, an abasic modification of gRNA (ØXØ) has been developed (Gu *et al.* 2026) by substituting positions 18 and 19 and extending up to position 22 (relative to the PAM position) with dSpacer (abasic deoxynucleotide lacking a 1′ hydroxyl group; Fig. 1A), a modification also used in RNA interference to avoid off-target effects (Lee *et al.* 2015, Park *et al.* 2018). As a biologically inspired approach, ØXØ outperforms pre-existing artificial strategies, including other gRNA modifications and Cas9 variants, in enhancing target specificity (Gu *et al.* 2026). Such benchmark studies have been conducted comprehensively using high-quality random mismatch target libraries (Fig. 1B and C), extensively sequenced to cover every putative off-target sequence (≤3MM), demonstrating that effects of ØXØ in gRNAs varied with positional sequence contexts (Gu *et al.* 2026). Therefore, to generalize the features learned from these high-quality datasets, we developed abCRISPR, a deep neural network (DNN) framework for the rational design of abasic gRNAs with minimized off-target activity, and applied it across all potent CRISPR-targetable sites in the human genome. abCRISPR provides a bioinformatics resource to ensure target specificity of abasic gRNA in genome editing, reducing concerns about off-target effects in experimental and clinical applications.

## 2 Implementation

We implemented abCRISPR, a DNN framework trained using exhaustive sequencing data, derived from comparative analysis of remaining random mismatch target libraries after *in vitro* CRISPR-Cas9-mediated cleavages (Fig. 1B–D). The training data covered the near-complete gRNA-DNA mismatch space (≤3MM, *n *= 97 583) for given gRNA sequences (*n *= 3; Fig. 1A and B, available as supplementary data at *Bioinformatics* online) with precisely quantified off-target activities, based on the extents of depletion (substrate abundance; SA) of “doped” synthetic DNA sequences (Fig. 1B and C, Fig. 1A, available as supplementary data at *Bioinformatics* online). Such informative few-shot training was conducted with paired datasets of abasic (ØXØ) and unmodified gRNAs (relative to no gRNA control; Fig. 1C and D). Particularly, these datasets provided ground-truth negatives, delineated with saturated sequencing depth, whereas conventional training datasets include spurious negatives confounded by signals below the detection limit (i.e. undetected mismatched genomic sites in the experiments). Of note, such a large number of diverse classes in few-shot learning has been shown to enhance model’s performance and generalizability while avoiding overfitting (Sbai *et al.* 2020, Wang *et al.* 2021).

A multilayer perceptron (MLP)-based multitask regression model was constructed to simultaneously predict SA values for abasic (ØXØ) and unmodified gRNAs (Fig. 1D). Mismatched sequences were encoded considering positional single-nucleotide contexts; using a substitution-based strategy, each nucleotide position, matched to a 20-nt-long gRNA spacer, was represented by a 10D vector capturing transition/transversion mismatches and one-hot encoded nucleotide compositions of both gRNA spacer and off-target sequences. Features were embedded into fully connected three hidden layers, considering the sizes of context features, positional dinucleotide context, nucleotide bases per position, and mismatch types (transition and transversion) per position.

Each layer was followed by rectified linear unit (ReLU) activation functions. To minimize overfitting, early stopping was implemented, halting training if the validation loss did not improve over 10 consecutive epochs. The mean squared error (MSE) loss function and the Adam optimizer (learning rate = 0.001) were used during training. The dataset was randomly partitioned into training (70%), validation (20%), and test (10%) sets and subjected to standard 10-fold cross-validation, which was used exclusively for benchmark evaluations (Fig. 1E–G, Fig. 1E and H, available as supplementary data at *Bioinformatics* online). For the final deployed model, sequential incremental training was conducted to ensure complete utilization of the full dataset. In this procedure, 10 iterations were performed, each alternately holding out a different 10% of the total data while the remaining 90% was used to progressively update model weights. During each iteration, training (70%) and validation (20%) sets were partitioned with different random seeds to further enhance model robustness and generalizability. The performance of the final deployed model was also independently assessed on experimentally derived off-target sequences from CIRCLE-seq (Tsai *et al.* 2017) and SITE-seq (Cameron *et al.* 2017) (Fig. 1H and I, Fig. 1J and K, available as supplementary data at *Bioinformatics* online).

After validating superior performance of abCRISPR in predicting gRNA with improved specificity (Fig. 1E–I), we applied the final deployed model to select highly specific ØXØ sequences from potent CRISPR-targetable sites in the human genome (CRISPOR efficiency > 55 and specificity > 50) (Haeussler *et al.* 2016) (Fig. 1J–L). The abCRISPR web server, implemented primarily using PHP and MySQL, provides information on the designed abasic gRNA sequences for the human genome (hg19; Fig. 1M and Fig. 2G–I, available as supplementary data at *Bioinformatics* online).

## 3 Results

In 10-fold cross-validation, abCRISPR accurately predicted SA values of off-target sites for both abasic (ØXØ) and unmodified gRNAs, closely matching their observed distributions (Fig. 1E), with strong correlation to experimental data (Pearson correlation coefficient; *r = *0.95–0.96). Since abCRISPR also predicted off-target activity of unmodified gRNAs, we compared this correlation value (Spearman correlation coefficient; *r = *0.95) with those from other deep learning-based methods that showed distinct prediction patterns (Fig. 1F), confirming its outperformance over CRISPR-Net (Lin *et al.* 2020), DeepCRISPR (Chuai *et al.* 2018), CRISPR-DIPOFF (Toufikuzzaman *et al.* 2024), CNN_std (Lin and Wong 2018), and CrisprDNT (Guan and Jiang 2023) (Spearman correlation coefficient; *r = *0.34, 0.17, −0.01, −0.02 and −0.13, respectively). In receiver operating characteristic (ROC) curve analysis, wherein off-targets were defined by an SA threshold (−2.17) derived by linear regression from a cleavage ratio of 0.5 in CRISPR-Cas9 experiments (Fig. 1C and D, available as supplementary data at *Bioinformatics* online), abCRISPR also surpassed the performance of other predictions in area under the curve (AUC = 0.98 versus 0.45–0.68; Fig. 1G). To provide a threshold-free comparison, we also evaluated recall for top-*k* experimentally validated off-targets (ranked by SA values) in the test set, against top-*k* predicted sites therein for 10-fold cross-validation (Fig. 1E, available as supplementary data at *Bioinformatics* online); abCRISPR showed the highest recall across all evaluated *k* values, outperforming the comparator methods (up to top 90%; 0.52–0.97 versus 0.01–0.89).

To assess generalizability regardless of gRNA sequences, we performed a leave-one-gRNA-out (LOO) evaluation after examining the distribution of SA values depending on gRNA sequences (Fig. 1F, available as supplementary data at *Bioinformatics* online). As T2 and EMX1 represent the lowest and highest SA value distributions, with overlap that covers the range of SA values observed in T3, we decided to use both T2 and EMX1 for training and T3 for testing to avoid the effects of differing SA value ranges in the training data (Fig. 1G, available as supplementary data at *Bioinformatics* online); abCRISPR showed superior performance over other methods (AUC = 0.78 versus 0.53–0.72), indicating the generalizability of its learning model even though it was trained on a limited number of gRNA sequences, when supplied with exhaustive mismatch sequences.

Independently of the training sets, we further evaluated the performance of the final deployed abCRISPR model, which was derived from sequential incremental training, using experimentally derived off-target sequences (≤6MM) from CIRCLE-seq (Tsai *et al.* 2017) with an optimized SA threshold determined by 10-fold cross-validation (−2.16, maximizing the F1 score; Fig. 1H and I, available as supplementary data at *Bioinformatics* online). As shown in the cumulative ratio of predicted off-targets (ranked according to read counts; Fig. 1H), abCRISPR robustly outperformed other deep-learning-based methods (developer-used threshold: 0.5) at every rank, even though they partly used this test set for training, exceeding them with sustained high positive predictive values (abCRISPR, CNN_std, CRISPR-DIPOFF, DeepCRISPR, CRISPR-Net, and CrisprDNT; average ratio: 0.63, 0.42, 0.26, 0.21, 0.02 and 0.00; total ratio: 0.53, 0.28, 0.14, 0.12, 0.00 and 0.00). Using high-confidence CIRCLE-seq data (Fig. 1I) that overlap with SITE-Seq (Cameron *et al.* 2017), similar results were observed for abCRISPR (average ratio: 0.60, 0.27, 0.22, 0.19, 0.02 and 0.02; total ratio: 0.49, 0.13, 0.12, 0.14, 0.01 and 0.01), indicating its superior accuracy and sensitivity. Only within the high ranks (3%–5%), CNN_std showed comparable positive predictive values to abCRISPR.

Most deep learning-based methods (except abCRISPR) were primarily trained using genome-wide sequencing results with detection limits (Kang *et al.* 2020, Zou *et al.* 2023) in the space of limited sequence complexity within the genome. Therefore, false positives and negatives in off-target prediction, including zero-shot inference, raise serious concerns due to spurious predicted negatives (mismatched sequences not detected in the experiments) in the training set (Guo *et al.* 2023), likely causing inferior performance even in these benchmarks that did not use predicted negatives. Of note, even though abCRISPR was only trained on sequences with ≤3MM, it showed generalized predictive power for CIRCLE-seq datasets that contain off-targets with >3MM (4–6MM; Fig. 1H and I). Indeed, abCRISPR showed superior performance on zero-shot inference when separately evaluated for 1–3MM and 4–6MM, particularly evident for only zero-shot cases (4–6MM) across different rank thresholds (Fig. 1J and K, available as supplementary data at *Bioinformatics* online).

Finally, we applied the final deployed abCRISPR model to a comprehensive set of highly efficient CRISPR-targetable sites (efficiency > 55, specificity > 50) in the human genome (61 761 658 gRNAs; Fig. 1J), initially selected using the CRISPOR program (Haeussler *et al.* 2016). Nevertheless, 99.1% of these gRNA sequences (*n *= 61 181 580) contain on average ∼12 off-target sites each across the genome (≤3MM; *n *= 747 063 488; Fig. 2A, available as supplementary data at *Bioinformatics* online). abCRISPR predicted that in 96.2% of cases there were significantly increased SA values for off-targets in abasic gRNAs (*n *= 58 875 004; Fig. 1K), when activities of off-target sites were compared relative to the corresponding unmodified gRNAs (*P *< .05, paired one-sided *t*-test as a per-gRNA threshold, wherein robustness to multiple-testing correction was confirmed; see Supplementary Methods, available as supplementary data at *Bioinformatics* online), showing an increased difference in SA values (ΔSA, relative to unmodified gRNAs; Fig. 1L). Among them, we experimentally validated reduced cleavage at 4 off-target sites for two abasic gRNAs (ØXØ) targeting VEGFA (*in vitro* CRISPR-Cas9 cleavage experiment; Fig. 2B–F, available as supplementary data at *Bioinformatics* online). Of note, the validation of the abasic gRNA prediction is supportive but experimentally (*n *= 2) and computationally limited (evaluated only for unmodified gRNAs). Although these results are useful as a proof of principle, users should be notified that this evidence is not yet sufficient for generalization and large-scale applicability, particularly for these very broad genome-wide claims. Overall, abCRISPR provides a comprehensive dataset of *bona fide* abasic gRNA (ØXØ) sequences with predicted enhancement of target specificity for editing the human genome (*n *= 58 875 004).

## 4 Functionality

Including implementation processes of abCRISPR with instructions, all Python codes and training sets are available on Zenodo (https://doi.org/10.5281/zenodo.20398246), enabling further modification of the model and the use of high-quality mismatch target library data for training other models. Results of abCRISPR for the human genome are available via the abCRISPR web interface (http://clip.korea.ac.kr/abCRISPR/). Users can search using two types of identifiers: gene symbol and genomic coordinates (hg19) with extended flanking regions (±0.5 or 1 kb), offering information on designed ØXØ sequences (Fig. 1M and Fig. 2G and H, available as supplementary data at *Bioinformatics* online; examples of the experimentally validated gRNAs targeting VEGFA) with averages of predicted SA values for off-target activity, their extents of mismatches, and statistical significance of differences in SA values of off-targets after ØXØ modification per gRNA (*P* value, paired one-sided *t*-test). Moreover, as descriptive effect-size metrics, mean ΔSA from the gRNA sequence (composite specificity-gain score; ΔSA_Mean_), ΔSA for the minimum SA value in the unmodified gRNA (improvement at the highest-risk off-target; ΔSA_HR_), and quartile rank of ΔSA_Mean_ (Q1–Q4) among statistically significant gRNAs (*P *< .05) were also provided (Fig. 2I, available as supplementary data at *Bioinformatics* online). Centered on the genomic coordinates or gene symbol of a given custom input, results of specific abasic gRNA sequences are returned in list form as a table providing links to the UCSC genome browser, enabling users to browse alongside other genome information.

In summary, here we present abCRISPR, a DNN framework based on training with high-quality mismatch target library data to accurately and sensitively evaluate off-target activity of abasic gRNA (ØXØ) compared with unmodified gRNA sequences. We also provide a comprehensive design of specific and potent abasic gRNA sequences predicted to perform with minimized off-target activity in the human genome. abCRISPR will support applications requiring high genome editing specificity, ensuring high on-target activity with the least off-target effects.

## Supplementary Material

btag568_Supplementary_Data

## Data Availability

abCRISPR data are available at https://doi.org/10.5281/zenodo.20398246 and http://clip.korea.ac.kr/abCRISPR/.

## References

[btag568-B1] Cameron P , Fuller CK, Donohoue PD et al Mapping the genomic landscape of CRISPR-Cas9 cleavage. Nat Methods 2017;14:600–6. 28459459 10.1038/nmeth.4284

[btag568-B2] Cho SW , Kim S, Kim JM et al Targeted genome engineering in human cells with the Cas9 RNA-guided endonuclease. Nat Biotechnol 2013;31:230–2. 23360966 10.1038/nbt.2507

[btag568-B3] Chuai G , Ma H, Yan J et al DeepCRISPR: optimized CRISPR guide RNA design by deep learning. Genome Biol 2018;19:80. 29945655 10.1186/s13059-018-1459-4 PMC6020378

[btag568-B4] Cong L , Ran FA, Cox D et al Multiplex genome engineering using CRISPR/Cas systems. Science 2013;339:819–23. 23287718 10.1126/science.1231143 PMC3795411

[btag568-B5] Doudna JA , Charpentier E. Genome editing. The new frontier of genome engineering with CRISPR-Cas9. Science 2014;346:1258096. 25430774 10.1126/science.1258096

[btag568-B6] Fu Y , Foden JA, Khayter C et al High-frequency off-target mutagenesis induced by CRISPR-Cas nucleases in human cells. Nat Biotechnol 2013;31:822–6. 23792628 10.1038/nbt.2623 PMC3773023

[btag568-B7] Gu D , Kim G-WD, Park M et al Abasic CRISPR RNAs inherently harness fidelity of SpCas9 for genome editing. Nat Chem Biol 2026. 10.1038/s41589-026-02139-8 41644849

[btag568-B8] Guan Z , Jiang Z. Transformer-based anti-noise models for CRISPR-Cas9 off-target activities prediction. Brief Bioinform 2023;24:bbad127. 10.1093/bib/bbad127 37068307

[btag568-B9] Guo C , Ma X, Gao F et al Off-target effects in CRISPR/Cas9 gene editing. Front Bioeng Biotechnol 2023;11:1143157. 36970624 10.3389/fbioe.2023.1143157 PMC10034092

[btag568-B10] Haeussler M , Schönig K, Eckert H et al Evaluation of off-target and on-target scoring algorithms and integration into the guide RNA selection tool CRISPOR. Genome Biol 2016;17:148. 27380939 10.1186/s13059-016-1012-2 PMC4934014

[btag568-B11] Hsu PD , Lander ES, Zhang F. Development and applications of CRISPR-Cas9 for genome engineering. Cell 2014;157:1262–78. 24906146 10.1016/j.cell.2014.05.010 PMC4343198

[btag568-B12] Hsu PD , Scott DA, Weinstein JA et al DNA targeting specificity of RNA-guided Cas9 nucleases. Nat Biotechnol 2013;31:827–32. 23873081 10.1038/nbt.2647 PMC3969858

[btag568-B13] Kang S-H , Lee W-J, An J-H et al Prediction-based highly sensitive CRISPR off-target validation using target-specific DNA enrichment. Nat Commun 2020;11:3596. 32681048 10.1038/s41467-020-17418-8 PMC7368065

[btag568-B14] Lee H-S , Seok H, Lee DH et al Abasic pivot substitution harnesses target specificity of RNA interference. Nat Commun 2015;6:10154. 26679372 10.1038/ncomms10154 PMC4703836

[btag568-B15] Lin J , Wong KC. Off-target predictions in CRISPR-Cas9 gene editing using deep learning. Bioinformatics 2018;34:i656–63. 30423072 10.1093/bioinformatics/bty554 PMC6129261

[btag568-B16] Lin J, Zhang Z, Zhang S et al CRISPR-Net: a recurrent convolutional network quantifies CRISPR off-target activities with mismatches and indels. Adv Sci 2020;7:1903562.

[btag568-B17] Lin Y , Cradick TJ, Brown MT et al CRISPR/Cas9 systems have off-target activity with insertions or deletions between target DNA and guide RNA sequences. Nucleic Acids Res 2014;42:7473–85. 24838573 10.1093/nar/gku402 PMC4066799

[btag568-B18] Mali P , Yang L, Esvelt KM et al RNA-guided human genome engineering via Cas9. Science 2013;339:823–6. 23287722 10.1126/science.1232033 PMC3712628

[btag568-B19] Park J , Ahn SH, Cho KM et al siAbasic: a comprehensive database for potent siRNA-6Ø sequences without off-target effects. Database (Oxford) 2018;2018:bay109. 10.1093/database/bay109 PMC6183071 30321353

[btag568-B20] Sander JD , Joung JK. CRISPR-Cas systems for editing, regulating and targeting genomes. Nat Biotechnol 2014;32:347–55. 24584096 10.1038/nbt.2842 PMC4022601

[btag568-B21] Sbai O , Couprie C, Aubry M. Impact of base dataset design on few-shot image classification. Img Proc Comp Vis Res 2020;12361:597–613.

[btag568-B22] Toufikuzzaman M , Hassan Samee MA, Sohel Rahman M. CRISPR-DIPOFF: an interpretable deep learning approach for CRISPR Cas-9 off-target prediction. Brief Bioinform 2024;25:bbad530. 10.1093/bib/bbad530 PMC10883906 38388680

[btag568-B23] Tsai SQ , Nguyen NT, Malagon-Lopez J et al CIRCLE-seq: a highly sensitive in vitro screen for genome-wide CRISPR-Cas9 nuclease off-targets. Nat Methods 2017;14:607–14. 28459458 10.1038/nmeth.4278 PMC5924695

[btag568-B24] Wang Y , Yao Q, Kwok JT et al Generalizing from a few examples: a survey on few-shot learning. ACM Comput Surv 2021;53:1–34.

[btag568-B25] Zhang X-H , Tee LY, Wang X-G et al Off-target effects in CRISPR/Cas9-mediated genome engineering. Mol Ther Nucleic Acids 2015;4:e264. 26575098 10.1038/mtna.2015.37 PMC4877446

[btag568-B26] Zou RS , Liu Y, Gaido OER et al Improving the sensitivity of in vivo CRISPR off-target detection with DISCOVER-Seq. Nat Methods 2023;20:706–13. 37024653 10.1038/s41592-023-01840-z PMC10172116

