# Supplementary material for: abCRISPR: deep learning-based design of abasic gRNA sequences for specific CRISPR-Cas9 genome editing
Source: Bioinformatics. 2026 Jul 29;42(8):btag568. doi: 10.1093/bioinformatics/btag568 (PMC13466718; doi:10.1093/bioinformatics/btag568)
Supplement: Supplementary file 1 [file btag568_supplementary_data.zip › abCRISPR_Supplementary_Figures.pdf]

Supplementary Figure 1

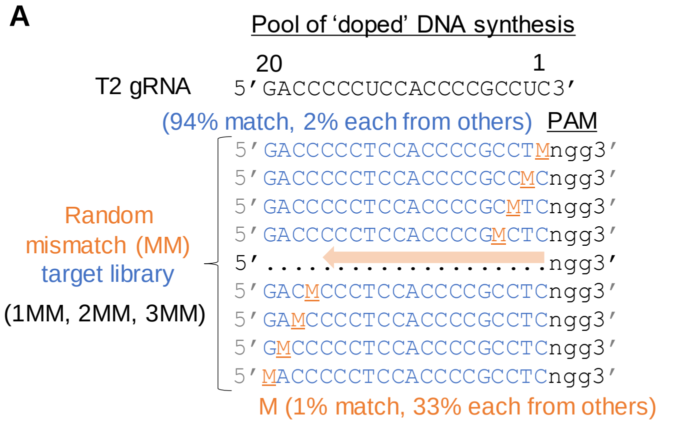

B

Number of combinations : *n* = 1 | *n* = 60 | *n* = 1,710 | *n* = 30,780

Sample (SRP number)	Type	Total reads	On-target	1MM	2MM	3MM	Spike-in
T2 (SRP455558)	Cont	26,971,314	169,231	4,005,952	7,055,900	6,087,470	562,744
	gRNA	27,329,397	36,685	1,133,342	2,917,689	4,826,330	2,856,264
	ØXØ	23,907,482	33,640	1,129,048	3,510,105	5,418,240	1,669,530
T3 (SRP455560)	Cont	30,353,912	274,881	10,027,271	9,708,271	4,305,683	370,746
	gRNA	39,184,924	202,674	8,337,037	10,290,674	6,409,353	2,874,193
	ØXØ	33,639,629	146,897	6,612,733	10,189,967	6,175,037	1,164,543
EMX1 (SRP455565)	Cont	33,974,884	152,925	6,964,681	10,212,062	7,048,000	694,026
	gRNA	35,606,044	119,407	5,763,984	9,101,247	7,199,922	1,969,057
	ØXØ	33,019,529	80,548	4,365,861	8,668,279	7,554,453	1,236,552

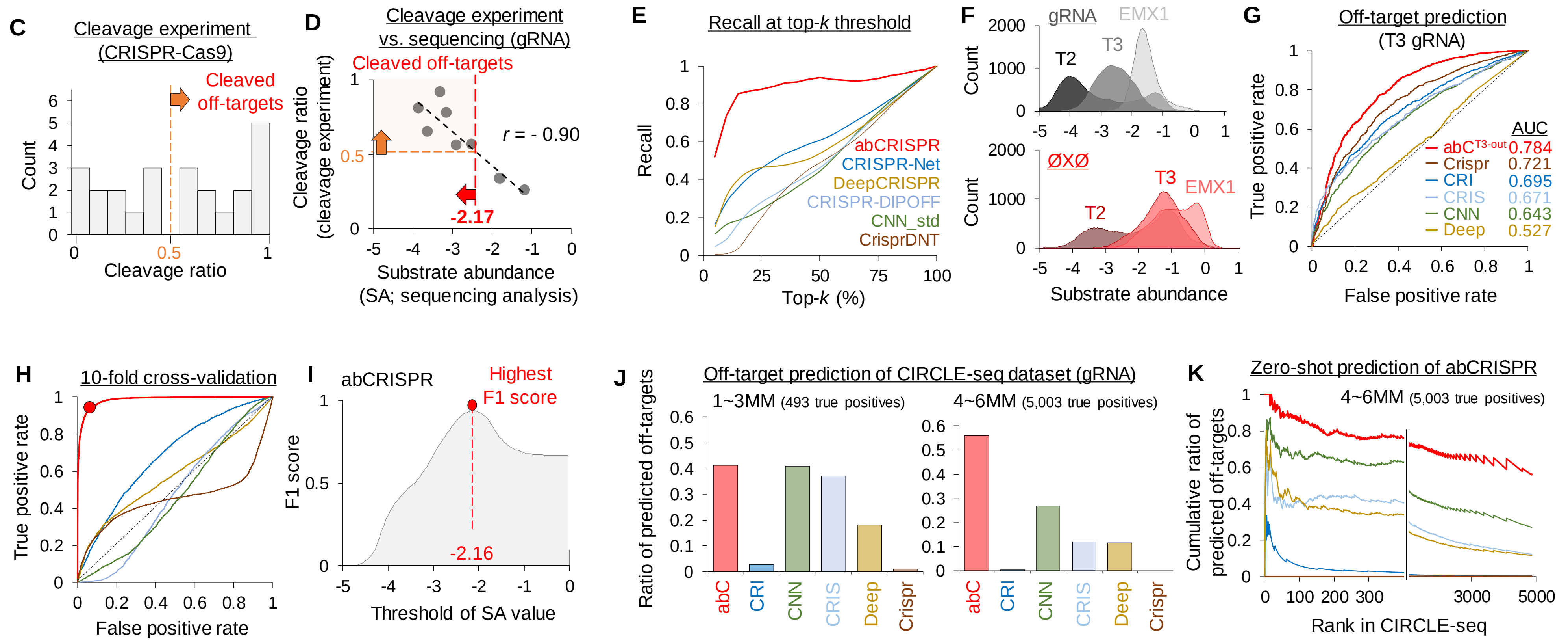

Supplementary Figure 1 | Use of random mismatch target libraries as training sets and determination of the threshold for off-target prediction in abCRISPR. (A) Schematic of the high-quality mismatch (MM) library constructed by pooling doped synthetic DNAs to generate comprehensive off-target substrates for *in vitro* CRISPR–Cas9 assays. For each position, the matched base was synthesized at high frequency (94%), with the three alternative bases doped at 2% each (denoted as blue), while a designated position (denoted as orange) was strongly randomized (1% matched base; 33% each alternative base) to ensure comprehensive representation of mismatched substrates. During *in vitro* CRISPR–Cas9 assays, an uncleavable spike-in control derived from a perfectly mismatched sequence for each target (the reverse sequence of the on-target) was added for normalization. The *VEGFA* T2 gRNA is shown as an example in this figure. **(B)** Sequencing summary of *in vitro* CRISPR-Cas9 assays using high-quality MM libraries for three gRNAs (T2, T3, and EMX1), including read depth for on-target, 1MM, 2MM, and 3MM substrates (unique sequence space: 1MM, *n* = 60; 2MM, *n* = 1,710; 3MM, *n* = 30,780), demonstrating near-exhaustive coverage of the gRNA–DNA mismatch space (≤3MM) used for abCRISPR model training and evaluation. **(C, D)** Experimental determination of the SA threshold for binary classification of off-targets in the high-quality mismatch dataset using individual *in vitro* cleavage assays. Previously reported *in vitro* CRISPR-Cas9 cleavage assays are summarized as a histogram of cleavage ratios (**C**; *n* = 24; on-targets and off-targets for unmodified gRNA and ØXØgRNA across three targets: T2, T3, and EMX1); targets with cleavage ratio ≥ 0.5 were defined as "cleaved off-targets". For off-targets of unmodified gRNA (**D**; *n* = 9), cleavage ratio was fitted against substrate abundance (SA) by linear regression and shown as a scatter plot; the SA value corresponding to a cleavage ratio of 0.5 (SA = −2.17) was used as the experimentally determined SA threshold for classifying cleaved off-targets; Pearson correlation coefficient, *r*. **(E)** Recall at varying top-*k* thresholds from 10-fold cross-validation. Positives are defined as the top-*k*% of experimentally validated off-target sites ranked by SA values; positive predictions are the top-*k*% of sites ranked by each model score. Recall is defined as the ratio of true positives / (true positives + false negatives), reflecting the ability of each model to identify experimentally confirmed off-target sites within the top-*k* predictions. **(F, G)** Leave-one-gRNA-out (LOO) evaluation of abCRISPR, trained on the mismatch target libraries of T2 and EMX1 gRNAs representing the low and high extremes of the SA value distribution among the three training gRNAs, respectively (**F**), and tested on the held-out T3 mismatch target library (**G**). Substrate abundance distributions of off-target sequences (≤3MM) for unmodified gRNA (**F**, upper panel) and ØXØgRNA (**F**, lower panel) for T2, T3, and EMX1. Receiver operating characteristic (ROC) curves for the LOO evaluation show that abCRISPR outperformed all five comparator methods in predicting off-target activity for the held-out T3; area under the curve (AUC) values (**G**). **(H, I)** Cross-validation–based selection of the final SA threshold for binary classification of off-targets in the high-quality mismatch dataset using the same dataset as in Fig. 1F. Receiver operating characteristic (ROC) curves are shown for abCRISPR and pre-developed deep-learning–based models (CRISPR-Net, DeepCRISPR, CRISPR-DIPOFF, CNN_std, and CrisprDNT) (**H**); the red dot marks the operating point with the highest F1 score (TPR, 0.9408; FPR, 0.0635; F1 score, 0.9386). The distribution of SA thresholds that maximize the F1 score is shown as a density plot (**I**); the red dot and dashed line indicate the highest F1 threshold (SA = −2.16). **(J, K)** Ratio of predicted off-target sites on the same CIRCLE-seq dataset as in Fig. 1H (1–3MM, 493 true positives; 4–6MM, 5,003 true positives) for abCRISPR and five comparator methods, shown separately for 1–3MM (J, left) and 4–6MM (J, right). Cumulative ratio of predicted off-targets in the 4–6MM is further dissected by read-count rank in CIRCLE-seq (K), confirming zero-shot generalizability of abCRISPR.

Supplementary Figure 2

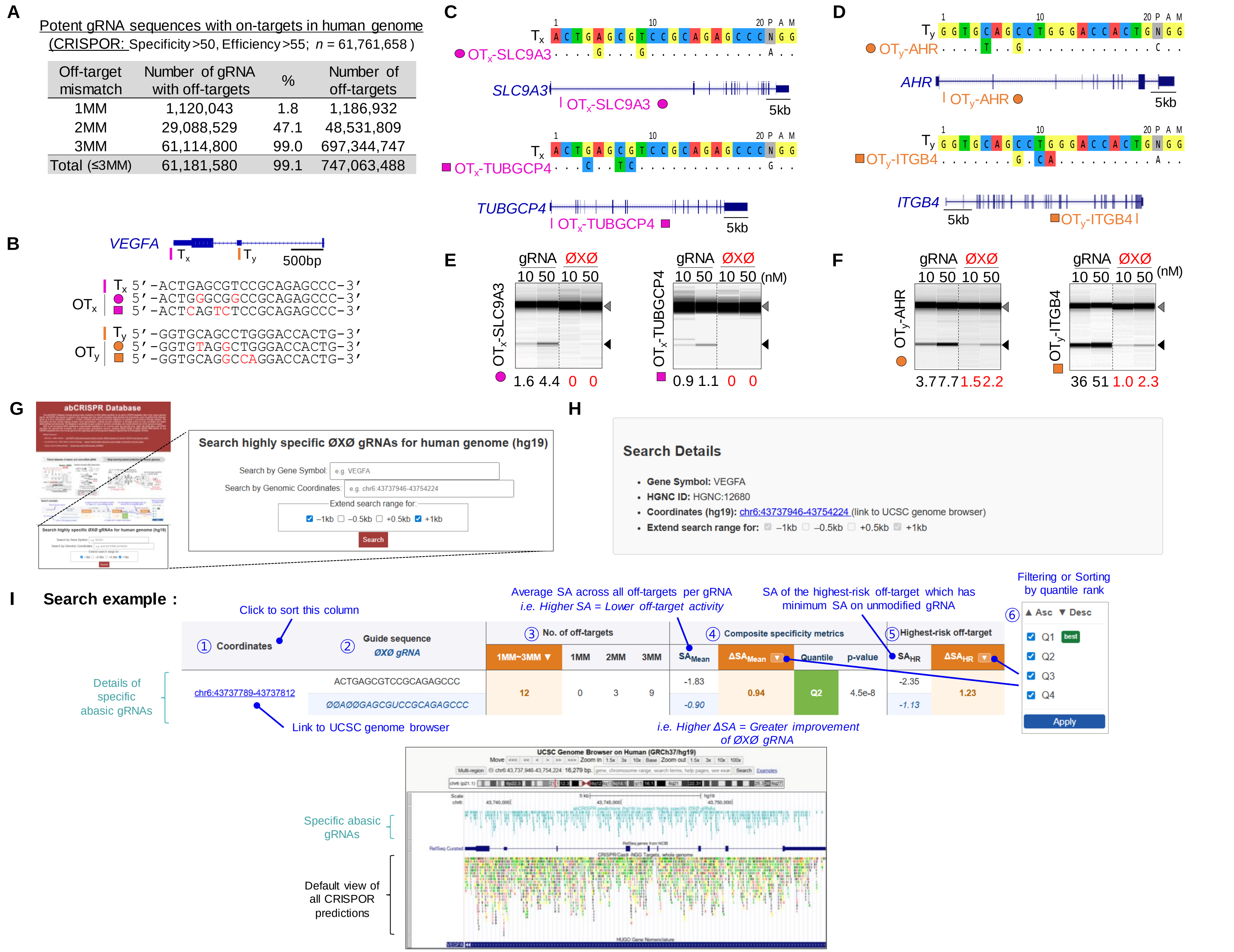

Off-target mismatch	Number of gRNA with off-targets	%	Number of off-targets
1MM	1,120,043	1.8	1,186,932
2MM	29,088,529	47.1	48,531,809
3MM	61,114,800	99.0	697,344,747
Total (≤3MM)	61,181,580	99.1	747,063,488

Supplementary Figure 2 | Experimental validation of abCRISPR predictions and overview of the web-interface. **(A)** Potent gRNA sequences in the human genome (n = 61,761,658; hg19), selected using CRISPOR (efficiency > 55, specificity > 50) and their off-target sequences (n = 747,063,488; ≤3MM). **(B-D)** Off-target sites of two selected *VEGFA*-targeting gRNAs (T_x and T_y; **B**) from abCRISPR, T_x (*SLC9A3* and *TUBGCP4*; **C**) and T_y (*AHR* and *ITGB4*; **D**), of which off-target activities were predicted to be significantly reduced by ØXØ modification; gRNA (spacer), upper sequence; off-target, lower sequence; gene structure in genome browser (lower graph) with indication of off-target sites (circle and rectangle; purple for T_x; yellow for T_y). **(E, F)** *in vitro* CRISPR–Cas9 assays with the off-target sites for T_x (**E**) and T_y (**F**). **(G)** Overview of the abCRISPR web interface, where users can search by gene symbol or genomic coordinates (hg19) with a user-selected flanking window (±0.5 or ±1 kb). **(H)** Search summary returned upon query, displaying the gene symbol, HGNC identifier, and genomic coordinates (hg19) linked to the UCSC Genome Browser, together with the selected search range options. **(I)** Representative output of the abCRISPR web interface, comprising a sortable and filterable results table (upper panel) and the corresponding UCSC Genome Browser view (lower panel). Each gRNA entry provides genomic coordinates (①, linking directly to the UCSC Genome Browser), target (②, upper row) and ØXØ gRNA (②, lower row, highlighted in blue) sequences, together with the total number of predicted off-target sites (③, ≤3MM, highlighted in orange) and its breakdown stratified by mismatch count (1MM, 2MM, and 3MM, respectively). Under the Composite specificity metrics section (④), the mean predicted SA across all off-target sites for unmodified (SA_{Mean}, upper row) and ØXØ gRNA (SA_{Mean}, lower row, highlighted in blue), the difference in SA between ØXØ and unmodified gRNA (ΔSA_{Mean}), a quantile rank of ΔSA_{Mean} among statistically significant gRNAs (Q1–Q4, where Q1 denotes the top 25% of specificity gain, filterable via dropdown), and a statistical significance value (*P* value, one-sided *t*-test) are provided. Under the Highest-risk off-target section (⑤), the minimum predicted SA among all off-targets of the unmodified gRNA reflecting the highest-risk site (SA_{HR}, upper row) and the predicted SA at that site for ØXØ gRNA (SA_{HR}, lower row), together with the ΔSA at that site (ΔSA_{HR}, filterable via dropdown), are displayed. Columns for ΔSA_{Mean} (④) and ΔSA_{HR} (⑤) are independently sortable and filterable by quartile rank via a dropdown menu (⑥). The linked UCSC Genome Browser view (lower panel) displays tracks for specific abasic gRNAs predicted by abCRISPR (cyan), CRISPOR predictions (gRNAs with specificity >50 and efficiency >55 are shown in green), and the RefSeq Curated gene model. Results are shown here for VEGFA as a representative example.
