# Supplementary material for: abCRISPR: deep learning-based design of abasic gRNA sequences for specific CRISPR-Cas9 genome editing
Source: Bioinformatics. 2026 Jul 29;42(8):btag568. doi: 10.1093/bioinformatics/btag568 (PMC13466718; doi:10.1093/bioinformatics/btag568)
Supplement: Supplementary file 1 [file btag568_supplementary_data.zip › Supplementary_Data.zip!/Supplementary_Data/abCRISPR_supplementary_method_072726_FF.docx]

**Supplementary Methods**

**Training dataset**

As the training dataset for abCRISPR, we used our high-quality sequencing results of random mismatch target libraries (≤ 3MM) from *in vitro* CRISPR-Cas9 cleavage experiments (Gu, et al., 2026). In this training data set, there are 97,583 mismatched sequences for 3 given gRNA sequences (spacer sequences; T2, GACCCCCTCCACCCCGCCTC; T3, GGTGAGTGAGTGTGTGCGTG; EMX1, GAGTCCGAGCAGAAGAAGAA) with quantified ratios relative to no gRNA control for abasic (ØXØ) gRNA and unmodified gRNA, used to calculate substrate abundance values (SA, log_2_ ratios). The training dataset is available at Zenodo (`dataset/ SA_3gRNAs_3MM_Q37.csv`, https://doi.org/10.5281/zenodo.20398246).

**Development of abCRISPR**

We developed a deep learning model using neural networks (DNN) for abCRISPR, trained with high-quality sequencing data comprising pairs of gRNA and off-target sequences (*n* = 97,583; ≤3MM) and their SA values for ØXØ gRNA and unmodified gRNA (*n* = 3; T2, T3, and EMX1). The training dataset was initially divided into 10 folds using scikit-learn (sklearn.model_selection.KFold; Python code: `code/split_fold.py`) to define the first fold as the test set (fold1 test set; 10%) for 10-fold cross-validation, and the remaining 90% (fold-1 training set) was then randomly partitioned into training (70%) and validation (20%) with `train_test_split` (sklearn.model_selection.train_test_split; Python code: `code/data_loader.py`). Off-target sequences were systematically encoded using a substitution-based strategy, where each nucleotide position, matched to a 20-nt-long gRNA spacer, was represented by a 10-dimensional vector capturing transition/transversion mismatches and one-hot encoded nucleotide compositions of both gRNA spacer and off-target sequences (*n* = 20 × (2 + 4 + 4) = 200 ; Python code : `code/encoder.py`).

A multilayer perceptron (MLP)-based multitask regression model was developed using PyTorch (torch.nn; Python code : `code/models.py`) to simultaneously predict two correlated continuous outputs: SA values for ØXØ and unmodified gRNA. The encoded input sequences were flattened into vectors of length 200, which were used as the input layer capturing positional single-nucleotide contexts. Subsequently, three fully connected hidden layers were arranged by rationally considering the sizes of context features, positional dinucleotide context (2-nt: *n* = 20 x 4 x 4 = 320 nodes), nucleotide bases per position (4-base: *n*= 20 x 4 = 80 nodes), and mismatch types (transition and transversion) per position (mismatch: *n* = 20 x 2 = 40 nodes). Each layer was followed by Rectified Linear Unit (ReLU) activation functions, with the final output layer producing predictions for SA values for ØXØ and unmodified gRNA. To minimize overfitting, early stopping was implemented, halting training if the validation loss did not improve over ten consecutive epochs. The mean squared error (MSE) loss function and the Adam optimizer with a learning rate of 0.001 were used during training (Python code : `code/train.py`).

Model performance was initially assessed via 10-fold cross-validation, followed by scatter plot analysis to evaluate correlations (Pearson correlation coefficients) between actual and predicted substrate abundances in both gRNA and ØXØ gRNA, confirming the model's predictive capability. Notably, the comprehensiveness of random mismatch target libraries, which cover all instances with ≤3MM, provided well-defined training sets that contributed to the model’s strong performance. Additionally, encoding mismatches into two statuses (transition and transversion) helped overcome limitations of the training dataset, which only included a limited number of gRNA sequences (T2, T3 and EMX1).

To develop the final abCRISPR models while avoiding reliance on a single split and maximizing training coverage, we implemented sequential incremental training over 10 iterations. In each iteration, every 10% of total data was alternately excluded, and the rest of the data were progressively updated to model weights carried over from the previous round (no re-initialization). During each iteration time, training (70%) and validation (20%) were partitioned with different random seeds to further enhance model robustness and generalizability. All deep learning operations and analyses were implemented in Python using PyTorch, scikit-learn, NumPy, and Pandas libraries, and the full code with instructions for reproducibility are available on Zenodo (<https://doi.org/10.5281/zenodo.20398246>).

**Comparison of deep-learning based off-target prediction models**

We evaluated the performance of abCRISPR in predicting off-target activity for unmodified gRNAs, comparing it with five pre-developed deep-learning–based models for off-target prediction: DeepCRISPR (Chuai, et al., 2018), CNN_std (Lin and Wong, 2018), CRISPR-Net (Lin, et al., 2020), CrisprDNT (Guan and Jiang, 2023), and CRISPR-DIPOFF (Toufikuzzaman, et al., 2024). For these benchmark studies, we used a test set of fold-1 in 10-fold cross-validation, derived from the random mismatch target libraries, to compare their predictions with the experimental results. The programs were run as follows: abCRISPR using the 10-fold cross-validation model (`models/SA_3gRNAs_3MM_Q37.fold1.pth` at https://doi.org/10.5281/zenodo.20398246), others with default model and parameters supplied from the repositories (DeepCRISPR: `trained_models/offtar_pt_cnn.tar.gz` at <https://github.com/bm2-lab/DeepCRISPR>; CNN_std: `CNN_std_model/model_cnn_v1.h5`, at <https://github.com/MichaelLinn/off_target_prediction>); CRISPR-Net: `code/scoring_models/CRISPR_Net_CIRCLE_elevation_SITE_weights.h5` at <https://github.com/JasonLinjc/CRISPR-Net>); CrisprDNT: `saved_model/encodedmismatchtype14x23Kleinstiver_5gRNAwithoutTsai.pkl+NCEandMAE_new_crispr_ip.h5` at <https://github.com/gzrgzx/CrisprDNT>; CRISPR-DIPOFF: `Trained_Models/best_lstm_model.pth` at <https://github.com/tzpranto/CRISPR-DIPOFF>). Of note, since DeepCRISPR requires epigenetic tracks for running, we chose the best-performing combination of epigenetic tracks (CTCF=1, Dnase=1, H3K4me3=1 and RRBS=1) among all possible combinations of parameters based on F1 score.

Off target activities predicted by the deep-learning–based models and derived from experimental data were compared for their correlation, wherein Spearman correlation coefficients were calculated using SciPy (<https://scipy.org/>, scipy.stats) and visualized as a pairwise correlation matrix with a heatmap using seaborn (<https://seaborn.pydata.org/>). Of note, for this correlation analysis, we used the negative values of SA from abCRISPR and experimental data to compare with off-target scores derived from other programs.

To define off-targets from the experimental SA values, we used -2.17 as a threshold because the value was derived from linear regression of cleavage ratio (0.5) in the previous CRISPR-Cas9 experiments (Gu, et al., 2026); details are provided in Supplementary Fig. S1C, D. Model performance under this binary classification for off-targets was evaluated using receiver operating characteristic (ROC) analysis in 10-fold cross-validation. The area under the ROC curve (AUC) was computed using scikit-learn (<http://scikit-learn.org/>, sklearn.metrics).

To provide a threshold-free evaluation, we assessed recall at varying top-*k* thresholds from 10-fold cross-validation as previously described (Haeussler, et al., 2016), with minor modifications. Positives were defined as the top-*k*% of experimentally validated off-target sites ranked by SA values, and positive predictions were the top-*k*% of sites ranked by each model's score in the test set. Recall was defined as the proportion of true positives among all positives (true positives + false negatives), reflecting the ability of each model to identify experimentally confirmed off-target sites within the top-*k* predictions. This analysis was performed across *k* values from 5% to 95%, with pairwise comparisons reported up to the top 90% (`threshold_free_eval/`, <https://doi.org/10.5281/zenodo.20398246>).

To assess cross-gRNA generalizability, we performed a leave-one-gRNA-out (LOO) evaluation. SA values for unmodified and ØXØ gRNAs across the three mismatch target libraries (T2, T3, EMX1) were binned at 0.05-unit intervals (numpy.histogram) and smoothed with a centered 5-bin uniform moving-average kernel (pandas.Series.rolling(window=5, center=True)) for visualization as overlaid area charts. As T2 and EMX1 represent the low and high extremes of the SA value distribution among the three gRNA sequences, respectively, and the SA values of T3 fall within the range spanned by T2 and EMX1, T3 was held out for testing while T2 and EMX1 were used for training to exclude the confounding effects of SA range differences on evaluation. The LOO model was trained on the combined mismatch target libraries of T2 and EMX1 (`models/T3out.pth`, <https://doi.org/10.5281/zenodo.20398246>) and evaluated on the held-out T3 dataset using the same binary classification threshold (SA = −2.17). The area under the ROC curve (AUC) was computed using scikit-learn (http://scikit-learn.org/, sklearn.metrics) and compared between abCRISPR and the five comparator methods. Of note, the outperformance of abCRISPR in this few-shot learning setting was achieved by using comprehensive, high-quality training data, which cover the near-complete gRNA-DNA mismatch space (≤3MM, n = 97,583).

To use independent experimental data of CRISPR-Cas9 off-targets for the validation of the final deployed model, we retrieved off-target sequences identified by CIRCLE-seq (Tsai, et al., 2017) after selecting those with ≤6MM, NGG PAM, no bulged mismatch, and off-target site length = 20 nt (*n* = 5,496) from the “list of all CIRCLE-seq detected off-target sites” (*n* = 7,374). Since experimentally derived off-target sequences can be considered as true positives (lacking reliable true negatives due to detection limits), we used this CIRCLE-Seq dataset to estimate true positive values of the prediction with sensitivity by calculating the cumulative fraction of predicted off-targets based on the rank of read counts in CIRCLE-Seq (ties of read count were resolved by descending model score). For this analysis, we used the final abCRISPR model (`models/SA_3gRNAs_3MM_Q37.iteration10.pth`, https://doi.org/10.5281/zenodo.20398246) and classified off-targets in abCRISPR prediction using an optimized SA threshold (-2.16), determined at the operating point corresponding to the maximum F1 score (true positive rate = 0.9408; false positive rate = 0.0635; F1 score = 0.9386) in 10-fold cross-validation. For other deep-learning–based models, we used 0.5 as a threshold in binary classification to define off-targets as suggested by the developers. The same analyses were performed with high-confidence CIRCLE-seq (*n* = 758), subsequently selected by overlapping the CIRCLE-Seq dataset (*n* = 5,496) for shared gRNA sequences (*n* = 2) with SITE-seq data (Cameron, et al., 2017), derived from "SITE-Seq target sites recovered” (*n* = 6,097; ≤6MM, NGG PAM, no bulged mismatch, and off-target site length = 20 nt). To further characterize zero-shot generalizability, we stratified the CIRCLE-seq validation set by mismatch count, separately computing the ratio of predicted off-target sites for the 1–3MM (*n* = 493 true positives) and 4–6MM (*n* = 5,003 true positives) subsets. For the zero-shot cases (4–6MM), the cumulative ratio of predicted off-targets was further dissected based on the rank of read counts in CIRCLE-seq, as described above. All datasets used for benchmarking were deposited to the Zenodo (`dataset/benchmark_dataset_with_predictions.xlsx`; https://doi.org/10.5281/zenodo.20398246).

**abCRISPR-based design of ØXØ sequences for human genome**

To predict potent gRNA sequences with significantly improved specificity through ØXØ modification, we applied abCRISPR to potent gRNA sequences (*n* = 61,761,658) selected from CRISPR-targetable sites in the human genome (CRISPOR (Haeussler, et al., 2016); efficiency > 55, specificity > 50). The potent gRNA sequences (“high predicted cleavage”) were retrieved from the “CRISPR Targets” track (track color = green) in the UCSC genome browser. Off-target coordinates corresponding to the selected gRNA sequences (excluding those with more than 300 off-target sites) were downloaded (<https://hgdownload.soe.ucsc.edu/gbdb/hg19/crisprAll/>) and used to extract off-target sequences (≤3MM; *n* = 747,063,488). SA values for both ØXØ and unmodified gRNAs were then predicted using the final deployed abCRISPR model (`models/SA_3gRNAs_3MM_Q37.iteration10.pth`, <https://doi.org/10.5281/zenodo.20398246>).

To statistically evaluate differences in predicted SA values between ØXØ and unmodified gRNA, the distribution of average SA values per gRNA (*n* = 61,181,580) for ØXØ was compared to those of unmodified gRNA using a paired one-sided *t*-test (scipy.stats.ttest_rel(alternative="less")), after confirming normality of paired differences via the Shapiro–Wilk test (median *W* = 0.924; scipy.stats.shapiro) on a random sample of 10,000 gRNAs. For visualization, 10% of observations were randomly sampled by reservoir sampling in Python (random.randint). Only statistically significant ØXØ sequences (*P* < 0.05, *n* = 58,875,004) were provided on the web server (<http://clip.korea.ac.kr/abCRISPR/>; hg19), implemented using MySQL. For each gRNA with statistically significant ØXØ improvement (*P* < 0.05), the following descriptive effect-size metrics were calculated and provided in the abCRISPR web interface. To quantify the overall improvement in off-target specificity per gRNA, mean predicted SA values across all off-target sites (≤3MM) were computed for unmodified (SA_Mean_, unmodified) and ØXØ gRNA (SA_Mean_, ØXØ), and their difference (ΔSA_Mean_) was used as a composite specificity-gain score reflecting the average improvement conferred by ØXØ modification. gRNAs were then ranked by ΔSA_Mean_ among all statistically significant gRNAs and assigned a quartile rank (Q1–Q4; Q1, top 25% greatest specificity gain; Q2, 50–75th percentile; Q3, 25–50th percentile; Q4, bottom 25% least gain), provided in the web interface as a filterable and sortable metric to enable rank-based gRNA prioritization. To further quantify the improvement at the highest-risk off-target site per gRNA, the minimum predicted SA value across all off-target sites of the unmodified gRNA was identified as the highest-risk (HR) off-target, and the predicted SA of the ØXØ gRNA at that same site (SA_HR_) and their difference (ΔSA_HR_) were calculated as a metric reflecting the improvement at the highest-risk off-target. Of note, as each *t*-test evaluates a single gRNA against its own set of off-target sites (≤3MM; average ~12 per gRNA), these *P* values represent per-comparison error rates that are appropriate to consult when selecting a single highly specific ØXØ gRNA and that are reported on the web interface without correction across gRNAs. To confirm the robustness of the genome-wide significant proportion, we separately applied the Benjamini–Hochberg (BH) procedure to the per-gRNA *P* values (*n* = 61,181,580) at a false discovery rate of *q* = 0.05 using statsmodels (https://www.statsmodels.org/, statsmodels.stats.multitest.multipletests(method="fdr_bh", alpha=0.05)), which retained 58,852,998 gRNAs (96.19%), only 22,006 fewer than the 58,875,004 gRNAs significant at the uncorrected threshold (96.23%), confirming that the proportion is robust to multiple-testing correction.

The web interface was developed using HTML, CSS, and JavaScript components, with enhanced functionality through the JQuery library (https://jquery.com/). For dynamic querying and retrieval, server-side scripting was implemented in PHP to handle asynchronous requests and access the backend MySQL database, ensuring efficient data retrieval and display. The server is hosted on a 24-core machine to ensure robust performance.

***In vitro* CRISPR-Cas9 cleavage experiments**

To experimentally validate the predicted ØXØ sequences, two ØXØ gRNA sequences (T_x_, ACTGAGCGTCCGCAGAGCCC; T_y_, GGTGCAGCCTGGGACCACTG) were selected from 11,153 CRISPR targetable sites based on the presence of their on-target sites within the VEGFA locus (from 500 bp upstream of exon 1 to exon 2) and off-target sites within other coding genes with ≤3MM. The selected off-targets (OT_x_-SLC9A3, ACTGGGCGGCCGCAGAGCCC; OT_x_-TUBGCP4, ACTCAGTCTCCGCAGAGCCC; OT_y_-AHR, GGTGTAGGCTGGGACCACTG; OT_y_-ITGB4, GGTGCAGGCCAGGACCACTG; mismatched bases were underlined) were subjected to *in vitro* CRISPR-Cas9 cleavage experiments.

Recombinant SpCas9 protein (EnGen Spy Cas9 NLS; New England Biolabs, #M0646) was used according to the manufacturer’s protocols with minor modifications. For the preparation of DNA substrates, target sequences were synthesized using custom synthesis services (Bionics, Korea). These sequences were designed to include adaptors commonly used in small RNA sequencing libraries (underlined: 5′-GTTCAGAGTTCTACAGTCCGACGATC-[target sequence]-TGGAATTCTCGGGTGCCAAGGC-3′). For *in vitro* SpCas9 cleavage assays for gRNAs (T_x_, T_y_), 100 nM SpCas9 protein (0.15 µL of 20µM stock) and 50 nM gRNA (0.075 µL of 20µM gRNA) were pre-incubated in 20 µL of 1.5X Cas9 Nuclease Reaction Buffer (NEB, #B0386) for 10 minutes at 25°C (prepared as a master mix for multiple samples with different substrates). Of note, 20 µM gRNA was prepared by annealing 2 µL of 100 µM synthetic crRNA and 2.2 µL of 100 µM tracrRNA (Sigma-Aldrich, #TRACRRNA05N) in 10 µL of ultra-pure water at 90°C for 4 min, ramped down to 25°C at -0.1°C/s for 10 minutes and 50 seconds, and stored at 4°C. The reaction mixture was then incubated with 10 nM DNA substrate in 30 µL of 1X Cas9 Nuclease Reaction Buffer at 37°C for 1 hour. To terminate the *in vitro* SpCas9 reaction, 1 µL of Proteinase K (20 mg/mL; Roche) was added and incubated at room temperature for 10 minutes. DNA from the reaction mixture was further purified using Oligo Clean & Concentrator kits (Zymo Research), and the cleaved products were analyzed using a Fragment Analyzer (Advanced Analytical Technologies), and cleavage rates were calculated as percentages.

**RNA synthesis and modification**

Custom synthesis and modification services from Bioneer (Korea) were used to produce crRNAs with abasic (ØXØ) modifications, the quality of which was monitored, reported, and confirmed by the companies through mass spectrometry. Abasic nucleotides (Ø) were introduced at the indicated positions in crRNA sequences, synthesized as dSpacer (abasic nucleotide with no hydroxyl group at the 1’position) as previously reported (Gu, et al., 2026). crRNAs selected by abCRISPR for experimental validation were synthesized, with spacer sequences underlined and 5′ phosphate denoted as 5′-p.; T_x_ (crRNA : 5'p-ACUGAGCGUCCGCAGAGCCCGUUUUAGAGCUAUGCUGUUUUG-3', ØXØ crRNA : 5'p-ØØAØØGAGCGUCCGCAGAGCCCGUUUUAGAGCUAUGCUGUUUUG-3'), T_y_ (crRNA : 5'p-GGUGCAGCCUGGGACCACUGGUUUUAGAGCUAUGCUGUUUUG-3', ØXØ crRNA : 5'p-ØØGØØGCAGCCUGGGACCACUGGUUUUAGAGCUAUGCUGUUUUG-3'). To generate synthetic gRNA (25 μM) as a complex of crRNA and tracrRNA, a mixture of synthetic crRNA (100 μM), tracrRNA (100 μM; Sigma-Aldrich, #TRACRRNA05N), and ultra-pure water (Biosesang) in a 1:1.1:1.9 volume ratio was annealed as follows: 90°C for 4 minutes, 70°C for 10 minutes, followed by 37°C for 15 minutes or ramped down to 25°C at -0.1°C/s for 10 minutes and 50 seconds, and stored at 4°C.

**References**

Cameron, P.*, et al.* Mapping the genomic landscape of CRISPR-Cas9 cleavage. *Nat Methods* 2017;14(6):600-606.

Chuai, G.*, et al.* DeepCRISPR: optimized CRISPR guide RNA design by deep learning. *Genome Biol* 2018;19(1):80.

Gu, D.*, et al.* Abasic CRISPR RNAs inherently harness fidelity of SpCas9 for genome editing. *Nat Chem Biol* 2026.

Guan, Z. and Jiang, Z. Transformer-based anti-noise models for CRISPR-Cas9 off-target activities prediction. *Brief Bioinform* 2023;24(3).

Haeussler, M.*, et al.* Evaluation of off-target and on-target scoring algorithms and integration into the guide RNA selection tool CRISPOR. *Genome Biol* 2016;17(1):148.

Lin, J. and Wong, K.C. Off-target predictions in CRISPR-Cas9 gene editing using deep learning. *Bioinformatics* 2018;34(17):i656-i663.

Lin, J.C.*, et al.* CRISPR-Net: A Recurrent Convolutional Network Quantifies CRISPR Off-Target Activities with Mismatches and Indels. *Adv Sci* 2020;7(13).

Toufikuzzaman, M., Hassan Samee, M.A. and Sohel Rahman, M. CRISPR-DIPOFF: an interpretable deep learning approach for CRISPR Cas-9 off-target prediction. *Brief Bioinform* 2024;25(2).

Tsai, S.Q.*, et al.* CIRCLE-seq: a highly sensitive in vitro screen for genome-wide CRISPR-Cas9 nuclease off-targets. *Nat Methods* 2017;14(6):607-614.
